# Incidence of autoimmune disease after hernia surgery with a mesh implant: national retrospective cohort study

**DOI:** 10.1093/bjs/znag025

**Published:** 2026-05-07

**Authors:** Maurits J C A M Gielen, Ahmed M Chaoui, Samantha Schoenmakers, Bas Vreugdenhil, Tim Lubbers, Richard P G Ten Broek, Rudi M H Roumen, Nicole D Bouvy, Willem A R Zwaans

**Affiliations:** Department of Surgery, Máxima Medical Centre, Veldhoven/Eindhoven, The Netherlands; GROW Research Institute for Oncology and Reproduction, Maastricht University, Maastricht, The Netherlands; Department of Surgery, Maastricht University Medical Centre, Maastricht, The Netherlands; Department of Surgery, Máxima Medical Centre, Veldhoven/Eindhoven, The Netherlands; Coöperatie VGZ, Arnhem, The Netherlands; Coöperatie VGZ, Arnhem, The Netherlands; GROW Research Institute for Oncology and Reproduction, Maastricht University, Maastricht, The Netherlands; Department of Surgery, Maastricht University Medical Centre, Maastricht, The Netherlands; Department of Surgery, Radboud University Medical Centre, Nijmegen, The Netherlands; Department of Surgery, Máxima Medical Centre, Veldhoven/Eindhoven, The Netherlands; SolviMáx, Centre of Expertise for Complex Groin and Abdominal Wall Pathology, Máxima Medical Centre, Eindhoven, The Netherlands; GROW Research Institute for Oncology and Reproduction, Maastricht University, Maastricht, The Netherlands; Department of Surgery, Maastricht University Medical Centre, Maastricht, The Netherlands; Coöperatie VGZ, Arnhem, The Netherlands; NUTRIM School of Nutrition and Translational Research in Metabolism, Maastricht UMC+, Maastricht, The Netherlands; Department of Surgery, Máxima Medical Centre, Veldhoven/Eindhoven, The Netherlands; SolviMáx, Centre of Expertise for Complex Groin and Abdominal Wall Pathology, Máxima Medical Centre, Eindhoven, The Netherlands; NUTRIM School of Nutrition and Translational Research in Metabolism, Maastricht UMC+, Maastricht, The Netherlands

## Abstract

**Background:**

Some patients report experiencing various systemic symptoms after surgical placement of a (polypropylene (PP)) mesh implant, including fatigue, arthritis, and myalgia. As these complaints could resemble systemic symptoms of autoimmune disease (AID), there are concerns that mesh implants could induce such disorders. The aim of this study was to identify the incidence of AID after mesh implantation, compared with controls in whom no mesh was implanted.

**Methods:**

In this national retrospective cohort study, approximately 4 million people insured with Dutch health insurer Coöperatie VGZ were screened for operative procedure reimbursements for inguinal and incisional hernia repair, between January 2015 and January 2019. Patients who underwent cholecystectomy were included as controls, as no mesh is implanted during this procedure. Patients were followed from 2 years before surgery to 3 years after surgery. The primary outcome was the difference in the incidence of AID after mesh implantation, compared with surgery without mesh. Propensity score matching for age, sex, and socioeconomic status was performed to reduce selection bias.

**Results:**

The cohort included 19 464 inguinal/incisional hernia patients (86.0% male) and 18 259 cholecystectomy patients (31.8% male). After matching, both groups consisted of 1500 patients. The incidence of new AID during follow-up was not statistically different between the two groups (1.1% for the intervention group and 1.5% for the control group; *P* = 0.520).

**Conclusion:**

Based on this cohort study, mesh implants do not increase the medium-term risk of developing AID.

## Teaser

A national retrospective cohort study on the incidence of autoimmune disease diagnoses after hernia surgery with mesh implants, compared with abdominal surgery without implants, as registered through operative procedure reimbursements. Groups were matched for relevant risk factors and the most common procedures before autoimmune disease diagnosis were identified.

## Introduction

Worldwide, >20 million inguinal, femoral, and abdominal hernia repairs are performed annually^1–4^. In the Netherlands, approximately 30 000 inguinal hernias are repaired every year, with inguinal hernia repair ranking among the most commonly performed surgeries^5^. In the past, reoperation was common due to the high risk of recurrence and the associated healthcare burden was high^6–8^. Since the introduction of polypropylene (PP) surgical mesh as reinforcement during hernia repair, recurrence rates have drastically dropped^6,9–12^. However, the introduction of PP meshes also introduced other complications, such as chronic pain and adhesion formation^10,13–15^. These complications are well documented and, as the use of mesh in repair procedures has increased from 85% to >95%, sufficient data are available to inform patients regarding the risks^16–20^. In recent years, concerns have been raised regarding another potential complication of mesh implantation: autoimmune syndrome induced by adjuvants (ASIA), also known as Shoenfeld’s syndrome^21^. Although rare, the syndrome could be a significant healthcare burden, as inguinal hernia repair is one of the most commonly performed surgeries^7,22,23^.

ASIA was first described by Shoenfeld and Agmon-Levin in 2011^21^ and was later updated by Watad *et al*. in 2017 and endorsed by Cohen Tervaert in 2018^24,25^. ASIA is characterized by a number of systemic complaints after implantation or injection of foreign material into the body; possible adjuvants include vaccines, prosthetics, breast implants, and PP mesh^26–30^. ASIA and autoimmune disease (AID) are associated with each other, are used interchangeably, and frequently coexist^24,25,29,31–33^. The increase in scientific publications and mainly the media coverage of ASIA has resulted in more patients advocating for mesh-free surgery, which, to date, is considered a suboptimal approach for treating most inguinal and incisional hernias^2^.

The aim of this study was to explore the incidence of AID (including potential ASIA) for a large, population-based, nationwide, retrospective cohort of patients. Patients who underwent inguinal or incisional hernia repair using (mainly PP) mesh (the intervention group) were compared with a cohort of patients who underwent cholecystectomy, a procedure that is frequently performed and for which no mesh implants are used (the control group)^24^. It was hypothesized that the presence of a mesh implant was not associated with an increased incidence of AID after adjusting for confounding variables.

## Methods

### Ethical approval

The use of individual anonymized data for research purposes was authorized by the Data Board of Coöperatie VGZ and falls under the National Health Insurance Act. The present study followed the declaration of Helsinki, no personal identification information was included in the data, and no patient informed consent or ethical approval was needed.

### Participants

Using Dutch healthcare insurance reimbursement data, patients aged >18 years with a newly registered procedure code for inguinal hernia repair, incisional hernia repair, or cholecystectomy between January 2015 and January 2020 were retrospectively identified (see *Table S1* for procedure codes). Relevant patient data and outcomes were recorded from January 2013 to January 2022 to allow 2 years of history before surgery and a minimum follow-up of 3 years after the procedure to ascertain the incidence of AID or ASIA after surgery. Patients were excluded if they changed healthcare insurance provider, received both interventions, or died due to an unrelated condition within the follow-up interval.

### Study design

A large, population-based, nationwide, retrospective cohort of patients was analysed using data from one of the four major healthcare insurance companies in the Netherlands: Coöperatie VGZ. Basic healthcare insurance is mandatory according to Dutch law, resulting in virtually all Dutch inhabitants being insured. All hospital care has to be reported to the corresponding healthcare insurance company as Diagnosis Treatment Combination (Dutch: DBC) codes or specific ‘procedure codes’ for surgical procedures. These codes are updated and adjusted by the Dutch Healthcare Authority (Dutch: NZa) annually. Diagnosis of AID was identified by first registration of any DBC codes related to AID (relevant codes were preselected; see *Table S1*). Within these codes, the DBC code for ‘Analysis for systemic disease without a diagnosis’ (code 03.13_020) was an indicator for referral to an immunologist, leading to no diagnosis or diagnosis of a non-specified AID, thus including ASIA. Most other common AID diagnoses have distinct codes and thus are declared under their distinct codes, increasing the specificity of code 03.12_020 for ASIA (*Table S1*).

Additionally, a separate analysis was performed wherein all patients with registered AID between 2017 and 2020 were screened for surgical procedures performed in the 2 years before their AID diagnosis. Procedures were ranked based on their incidence to identify whether inguinal/incisional hernia repairs were over-represented in the years before AID diagnosis, compared with cholecystectomy and other operative procedures.

### Interventions and covariates

Patients who underwent inguinal or incisional hernia repair formed the intervention group. Use of mesh is virtually standard procedure for all such repairs in the Netherlands, as recommended in (inter)national guidelines, with PP being the most frequently used material for mesh implants^1,17,18,20^. Procedure codes for other hernia surgeries (that is umbilical, epigastric/ventral, and congenital hernia surgeries) were excluded due to the increased ratio of mesh-free repairs for these surgeries. Patients who underwent a cholecystectomy were used as a control group. Confounding factors for AID that were extracted included sex, age, and socioeconomic status (SES) based on geographics^34^. SES was measured by generalized zip code via SES-WOA (SES based on wealth, educational attainment, and labour market participation), scoring between −7 and 3.1 (with education, wealth, and employment history contributing −2.2 to +1.1, −2.1 to +1.2, and −2.7 to +0.8 points respectively)^35^. Ethnicity and race were not used as covariates, in accordance with European law, as a scientific basis for their current relevance is absent.

### Outcomes

The primary outcome was the incidence of AID in reimbursement data in the 3 years after surgery, comparing (PP) mesh inguinal and incisional hernia repair with a (mesh-free) cholecystectomy group. The incidence of new AID alongside pre-existing AID was included. The secondary outcome included the presence of the DBC code 03.13_020 (‘Analysis for systemic disease without a diagnosis’).

For the separate analysis of the most common surgical procedures performed in the 2 years before developing AID, the incidence of each procedure was determined and ranked. As this backwards analysis did not allow for correction of possible confounders, the results for the identified population were compared with those for the general adult population without known AID to indicate differences in incidence.

### Statistical methods

Descriptive statistics are used to summarize the baseline characteristics. Differences in demographic data were analysed using Fisher’s exact test for nominal and ordinal data, and Wilcoxon’s rank-sum test for continuous data. Significant differences in baseline variables and possible confounders were corrected for using propensity score matching (PSM), aiming for all confounders to be far from significant with *P* ≥ 0.5, with PSM being repeated ten times to reduce systemic bias. A difference in the incidence of AID after PSM was tested for using Fisher’s exact test. Data are presented as *n* (%) or mean(s.d.), as appropriate. *P* < 0.05 was considered statistically significant. All analyses were performed using R, including the MatchIt package for PSM.

## Results

Approximately 4 million Dutch inhabitants were screened for eligibility. After applying inclusion and exclusion criteria, a total of 37 723 patients were identified. The intervention group consisted of 19 464 patients, with 86.0% being male, a mean(s.d.) age of 61.5(14.1) years, a mean(s.d.) SES score of −0.05(2.04), and an incidence of AID before surgery of 2.3%. The control group consisted of 18 259 patients, with 31.8% being male, a mean(s.d.) age of 54.5(15.7) years, a mean(s.d.) SES score of −0.10(2.04), and an incidence of AID before surgery of 3.1%. Patient characteristics and results after inclusion, with between-group comparisons, are summarized in *Table 1*. The incidence of new AID during the 3-year follow-up for the complete cohort was 1.5%. All of the covariates that were analysed were significantly different between the groups (*P* < 0.001).

**Table 1:** Patient characteristics and results after inclusion

	Total, *n* = 37 723	Hernia repair (with mesh), *n* = 19 464	Cholecystectomy (without mesh), *n* = 18 259	P*
Male	22 547 (59.8)	16 746 (86.0)	5801 (31.8)	<0.001†
Age (years), mean(s.d.)	58.10(15.31)	61.50(14.13)	54.49(15.69)	<0.001†
SES score, mean(s.d.)	−0.072(2.04)	−0.049(2.04)	−0.096(2.04)	<0.001†
**AID before surgery**				<0.001†
Absent	36 710 (97.3)	19 008 (97.7)	17 702 (96.9)	
Present	1013 (2.7)	456 (2.3)	557 (3.1)	
**AID after surgery**				<0.001†
Absent or pre-existing	37 155 (98.5)	19 210 (98.7)	17 945 (98.3)	
New	568 (1.5)	254 (1.3)	314 (1.7)	

Values are *n* (%) unless otherwise indicated. *Fisher’s exact test for nominal and ordinal data, and Wilcoxon’s rank-sum test for continuous data. †Statistically significant. SES, socioeconomic status; AID, autoimmune disease.

Patient characteristics and results after PSM, with between-group comparisons, are summarized in *Table 2*. After PSM, 1500 patients per group remained, with 85.0% being male and a mean(s.d.) age of 61.2(14.4) years. The incidence of new AID during follow-up was 1.1% for the mesh group, compared with 1.5% for the cholecystectomy group. Thus, no difference in the incidence of new AID during follow-up was demonstrated (*P* = 0.520). Within the limited group of patients with new AID after surgery, the percentage who had a claim for ‘Analysis for systemic disease without or with a non-specified AID diagnosis’ was approximately 2% and was therefore too small for statistical testing.

**Table 2:** Patient characteristics and results after PSM

	Total, *n* = 3 000	Hernia repair (with mesh), *n* = 1500	Cholecystectomy (without mesh), *n* = 1500	P*
Male	2550 (85.0)	1275 (85.0)	1275 (85.0)	1.000
Age (years), mean(s.d.)	61.22(14.39)	61.12(14.43)	61.31(14.35)	0.803
SES score, mean(s.d.)	−0.072(1.92)	−0.055(1.89)	−0.089(1.95)	0.516
**AID before surgery**				0.573
Absent	2949 (98.3)	1477 (98.5)	1472 (98.1)	
Present	51 (1.7)	23 (1.5)	28 (1.9)	
**AID after surgery**				0.520
Absent or pre-existing	2961 (98.7)	1483 (98.9)	1478 (98.5)	
New	39 (1.3)	17 (1.1)	22 (1.5)	

Values are *n* (%) unless otherwise indicated. *Fisher’s exact test for nominal and ordinal data, and Wilcoxon’s rank-sum test for continuous data. PSM, propensity score matching; SES, socioeconomic status; AID, autoimmune disease.

For the separate analysis of the most common procedures performed before AID diagnosis, the results are summarized in *Table 3* (see *Table S2* for the full list). Most notably, procedures to treat inflammatory/(pre)malignant dermal conditions and conditions related to increased age were strongly represented and were the most commonly performed procedures. In the AID population, cholecystitis treatment was the 9th most performed procedure, compared with inguinal hernia repair, which was the 18th most performed procedure (0.65% and 0.50% respectively). This illustrates that inguinal/incisional hernia repair was not disproportionally represented before AID diagnosis compared with other frequently performed procedures.

**Table 3:** Ranking of the ten most common procedures performed before AID diagnosis (adapted from *Table S2*)

Diagnosis related to procedures	Specialty_diagnosis	Within the AID population		Within the general adult population	
		Rank	Incidence (%)	Rank	Incidence (%)
Cataract	03.01_554	1	3.62	2	1.03
Malignant dermatosis	03.10_014	2	2.84	1	1.14
Premalignant dermatosis	03.10_017	3	2.00	3	0.66
Inflammatory dermatosis	03.10_013	4	1.68	33	0.08
After-cataract/posterior capsular opacification	03.01_557	5	1.31	5	0.34
Knee osteoarthritis	03.05_1801	6	1.07	6	0.22
Osteoarthritis pelvis/hip/upper leg	03.05_1701	7	0.84	7	0.20
Guidance delivery pregnancy, other	03.07_B41	8	0.69	4	0.40
Cholecystitis/cholelithiasis	03.03_323	9	0.65	10	0.18
Carpal tunnel syndrome (CTS), decompression CTS	03.04_351	10	0.63	19	0.11
Inguinal/femoral hernia	03.03_121	18	0.50	11	0.17

AID, autoimmune disease.

## Discussion

This study compares surgery using mesh with surgery not using mesh regarding the subsequent incidence of AID. Using a population-based approach, 3 years after surgery, the incidence of AID was comparable. Furthermore, the prevalence of referrals to immunologists for patients without a distinct immunological diagnosis (possibly including ASIA) or non-specific AID was marginal and comparable for both groups. The findings of the present study confirm the hypothesis that use of mesh implants in surgery is not associated with an increased risk of development of AID and the use of mesh should not be discouraged for fear of inducing AID.

With the increase in news reports and publications about mesh and autoimmunity, or ASIA, several systematic reviews have been performed^33,36–39^. However, these reviews are limited by the available evidence, as predominantly case series have been previously published^31–33,40^. Few studies of considerable quality and sample size have been published. Chughtai *et al*.^41^ performed a study that was largely similar to the present study and found no association between AID and mesh implantation when compared with colonoscopy. Another study by Chughtai *et al*.^42^, in which vaginal mesh implantation was assessed in 2102 patients as a potential inducer of AID, also found no association. However, in both of these studies, patients wo underwent surgery were compared with those who did not, which might be a limitation, considering that surgery generally leads to an inflammatory state^43,44^. Moreover, in these studies, AID was only registered when it was recorded by surgeons and not by immunologists or other relevant medical specialists, likely resulting in underestimates. The present study reliably overcomes these limitations by comparing surgery using mesh with surgery not using mesh and by including AID diagnoses registered by all medical specialists, including immunologists and rheumatologists.

Based on the theoretical hypothesis of ASIA, surgical procedures with implants should have higher incidences of AID. By ranking and sorting procedures, the present study found that inguinal hernia repair does not head the list of commonly performed surgeries before AID diagnosis at all. As most of the ASIA criteria can arise after a non-specific trigger, a causal relationship between a systemic symptom and the presence of previously implanted mesh is challenging as long as objective diagnostic tests (including blood analysis) are lacking^32,33,45,46^. Attributing AID to the implantation of a mesh is likely due to the frequent co-occurrence: high numbers of hernia repairs are performed annually and thus have been performed more often when AID develops. This falsely leads to the assumption of a causal relationship^47^.

The organization of the healthcare system in the Netherlands, as well as the specific conditions of the population insured by Coöperatie VGZ, strengthen the methodology of the present study. In the Netherlands, uninsured healthcare is virtually non-existent and accessibility to healthcare is mostly equal between the rich and the poor due to a fee-for-service system^48,49^. The data collected by the healthcare insurance company include a complete list of procedures and diagnoses for every individual, for all specialties, making missing diagnoses unlikely. Coöperatie VGZ had a market share of 23.6% of the population in 2022, with insurance holders across the entire country^15,50^. It should be noted that only 6.2–8.5% of inhabitants change their healthcare insurance provider (at the end of the year)^15^. Therefore, the analysed cohort should adequately represent the Dutch population. Additionally, by adopting a follow-up interval of 3 years, the aim was to include sufficient time for AID to develop and to bridge any subsequent delayed diagnosis of AID.

This study has several limitations of importance. First—considering the relevance of confounding factors to AID risk—BMI, smoking, age, sex, and environmental exposure were planned to be included to later adjust for their influence on the results^34^. However, the database that was used did not include data on BMI or smoking behaviour, due to privacy regulations. To partially account for their influence on the data, the SES score was used to assess possible unhealthy environmental exposure and behaviour^47^. Furthermore, as an ICD-10 code for ASIA is absent, no specific DBC code exists for ASIA either, therefore making the presently used code 03.13_020 a surrogate for ASIA and other non-specified AID. Conversely, to counteract this limitation, the present study included all AID DBC codes in the analysis to ensure potential ASIA candidates were not omitted and to include all theoretical chronic diseases secondary to inflammation caused by mesh implants. Lastly, although Dutch guidelines strongly advise hernia surgeons to perform inguinal and incisional hernia repairs (such as those in the present study) with PP mesh (90–95% of repairs), both the exact percentage of meshes being PP (*versus* polytetrafluoroethylene (PTFE) and polyester) and percentage of mesh-free tissue repairs are unknown. This leads to a small overestimate of the percentage of PP mesh-based hernia repairs in the intervention group, which could have had a theoretical impact if the incidence of AID in the intervention group was higher than in the control group, not vice versa.

Despite the assumptions, of which there are several, within the present study, the results suggest that the need for future, prospective, long-term studies on development of AID after mesh implantation is evidently not as urgent as previously thought. Prospective studies should focus on natural progression of complaints after mesh implantation, combined with objective findings and potential diagnostic modalities to verify actual autoimmunity. Nonetheless, the present study decreases the knowledge gap on the supposed causal relationship between AID (or potentially ASIA) and (PP) mesh in hernia repair surgery.

## Supplementary Material

znag025_Supplementary_Data

## Data Availability

Data will not be made publicly available, but, upon reasonable request, to test specific research questions, the authors are willing to collaborate. This research was not preregistered with a prior analysis plan.
