# Supplementary material for: Incidence of autoimmune disease after hernia surgery with a mesh implant: national retrospective cohort study
Source: Br J Surg. 2026 May 7;113(5):znag025. doi: 10.1093/bjs/znag025 (PMC13151888; doi:10.1093/bjs/znag025)
Supplement: znag025_Supplementary_Data [file znag025_supplementary_data.docx]

# Incidence of autoimmune disease after hernia surgery with a (polypropylene) mesh implant: national retrospective cohort study

Maurits J.C.A.M. Gielen^1,2,3*^, Ahmed M. Chaoui^1^, Samantha Schoenmakers^4^, Bas Vreugdenhil^4^, Tim Lubbers^,2,3^, Richard P.G. Ten Broek^5^, Rudi M.H. Roumen^1,6^, Nicole D. Bouvy^2,3,4,7^, Willem A.R. Zwaans^1,6,7^

Author affiliations

1 Department of Surgery, Máxima Medical Centre, Veldhoven/Eindhoven, The Netherlands

2 GROW Research Institute for Oncology and Reproduction, Maastricht University, Maastricht, The Netherlands.

3 Department of Surgery, Maastricht University Medical Centre, Maastricht, The Netherlands

4 Coöperatie VGZ, Arnhem, The Netherlands.

5 Department of Surgery, Radboud University Medical Centre, Nijmegen, The Netherlands.

6 SolviMáx, Centre of Expertise for Complex Groin and Abdominal Wall Pathology, Máxima Medical Centre, Eindhoven, The Netherlands.

7 NUTRIM School of Nutrition and Translational Research in Metabolism, Maastricht UMC+, Maastricht, The Netherlands.

Corresponding author*

Maurits J.C.A.M. Gielen MD, [maurits-jan.gielen@maastrichtuniversity.nl](mailto:maurits-jan.gielen@maastrichtuniversity.nl)

ORCID: 0000-0003-4787-4461

Postal address: GROW Research Institute for Oncology and Reproduction, Maastricht University, PO Box 616, 6200 MD, Maastricht, the Netherlands

**Supplementary Materials - Index**

| **Supplementary Figures and Tables** |  |
| --- | --- |
| Table S1. List of Diagnosis Treatment Codes (DBC codes) from multiple specialties to signal positive diagnosis of autoimmune disease | *page 3-4* |
| Table S2. Ranking of the most performed procedures prior to AID diagnosis compared to general population | *page 5* |

**Supplementary Tables**

**Table S1. List of Diagnosis Treatment Codes (DBC codes) from multiple specialties to signal positive diagnosis of autoimmune disease.**

| **Diagnosis** | **Specialty code** | **Diagnosis code** |
| --- | --- | --- |
| Eczema, constitutional | 03.10 | 005 |
| Eczema, seborrheic | 03.10 | 007 |
| Inflammatory dermatoses (pemphigus vulgaris) | 03.10 | 013 |
| Psoriasiform dermatoses (psoriasis) | 03.10 | 020 |
| Analysis systemic disease without diagnosis | 03.13 | 020 |
| Graves' Disease | 03.13 | 208 |
| Sarcoidosis | 03.13 | 501 |
| Granulomatous inflammation, nos | 03.13 | 502 |
| Behçet's Disease | 03.13 | 503 |
| Temporal arteritis, polymyalgia rheumatica | 03.13 | 506 |
| Psoriatic arthritis | 03.13 | 512 |
| Gout, crystal arthropathy, chondrocalcinosis | 03.13 | 513 |
| Bechterew's Disease/Ankylosing spondylitis | 03.13 | 515 |
| Fibromyalgia | 03.13 | 519 |
| Rheumatoid arthritis | 03.13 | 521 |
| Systemic lupus erythematosus/MTCD | 03.13 | 522 |
| Polymyositis | 03.13 | 523 |
| Sjogren’s Disease | 03.13 | 524 |
| Systemic sclerosis/CREST | 03.13 | 525 |
| Vasculitis (systemic) | 03.13 | 526 |
| Allergic vasculitis/Henoch-Schönlein | 03.13 | 527 |
| Guidance immunosuppressive treatment | 03.13 | 531 |
| Other systemic diseases, vasculitis nos | 03.13 | 599 |
| Other lung diseases nos (e.g. Goodpasture) | 03.13 | 609 |
| Pernicious anemia | 03.13 | 702 |
| Acquired hemolytic anemia | 03.13 | 706 |
| ITP/autoimmune thrombocytopenic purpura | 03.13 | 721 |
| Celiac's Disease | 03.13 | 920 |
| Crohn's Disease | 03.13 | 922 |
| Ulcerative Colitis | 03.13 | 923 |
| Autoimmune hepatitis | 03.13 | 943 |

**Table S1. List of Diagnosis Treatment Codes (DBC codes) from multiple specialties to signal positive diagnosis of autoimmune disease (continued).**

| **Diagnosis** | **Specialty code** | **Diagnosis code** |
| --- | --- | --- |
| Rheumatoid arthritis | 03.24 | 101 |
| Psoriatic arthritis (esp. peripheral) | 03.24 | 102 |
| Palindromic Rheumatism | 03.24 | 113 |
| Still's Disease, Adult onset | 03.24 | 114 |
| Ankylopoetic spondylitis | 03.24 | 201 |
| Psoriatic arthritis (esp. axial) | 03.24 | 202 |
| Systemic lupus erythematosus (SLE) | 03.24 | 301 |
| Cutaneous LE | 03.24 | 302 |
| Lupus like/antiphospholipid syndrome | 03.24 | 303 |
| CREST syndrome | 03.24 | 304 |
| Scleroderma | 03.24 | 305 |
| Mixed connective tissue disease (MCTD) | 03.24 | 306 |
| Poly-/Dermatomyositis | 03.24 | 307 |
| Sjögren's Disease | 03.24 | 308 |
| Polymyalgia rheumatic (PMR) | 03.24 | 309 |
| Temporal arteritis | 03.24 | 310 |
| Polyarteritis nodosa (PAN)/Microscopic polyangiitis | 03.24 | 311 |
| Wegener's Disease/Granulomatosis with polyangiitis | 03.24 | 312 |
| Sarcoidosis | 03.24 | 313 |
| Erythema nodosum | 03.24 | 314 |
| Amyloidosis (primary/secondary) | 03.24 | 315 |
| Behçet's Disease | 03.24 | 317 |
| Takayasu's Disease | 03.24 | 318 |
| Churg-Strauss Syndrome | 03.24 | 319 |
| Other vasculitis/systemic diseases | 03.24 | 399 |
| Raynaud's phenomenon | 03.24 | 716 |
| Psoriasis | 03.24 | 720 |
| Polymyalgia rheumatica/temporal arteritis | 03.30 | 313 |
| Amyotrophic lateral sclerosis (ALS) | 03.30 | 522 |
| Multiple sclerosis (MS) | 03.30 | 531 |
| Myasthenia gravis and myasthenic syndromes | 03.30 | 911 |

**Table S2. Ranking of the most common procedures performed before AID diagnosis compared to the general population.**

|  |  | Within the AID population | | Within the general adult population | |
| --- | --- | --- | --- | --- | --- |
| **Diagnosis related to procedures** | **Specialty_diagnosis** | Rank | Incidence | Rank | Incidence |
| Cataract | 03.01_554 | 1 | 3.62% | 2 | 1.03% |
| Malignant dermatosis | 03.10_14 | 2 | 2.84% | 1 | 1.14% |
| Premalignant dermatosis | 03.10_17 | 3 | 2.00% | 3 | 0.66% |
| Inflammatory dermatosis | 03.10_13 | 4 | 1.68% | 33 | 0.08% |
| After-cataract/Posterior capsular opacification | 03.01_557 | 5 | 1.31% | 5 | 0.34% |
| Knee osteoarthritis | 03.05_1801 | 6 | 1.07% | 6 | 0.22% |
| Osteoarthritis pelvis/hip/upper leg | 03.05_1701 | 7 | 0.84% | 7 | 0.20% |
| Guidance delivery pregnancy, other | 03.07_B41 | 8 | 0.69% | 4 | 0.40% |
| Cholecystitis/cholelithiasis | 03.03_323 | 9 | 0.65% | 10 | 0.18% |
| Carpal tunnel syndrome, decompression CTS | 03.04_351 | 10 | 0.63% | 19 | 0.11% |
| Menstrual cycle "disorders" | 03.07_G11 | 11 | 0.63% | 14 | 0.16% |
| Hemorrhoids | 03.03_117 | 12 | 0.62% | 8 | 0.20% |
| Diagnosis not specified | 03.10_27 | 13 | 0.61% | 40 | 0.07% |
| Chronic neurogenic lower back pain | 03.89_076 | 14 | 0.60% | 12 | 0.17% |
| Ganglion, large lipoma, unguis incarnatus | 03.03_170 | 15 | 0.53% | 13 | 0.16% |
| Naevi (all forms) | 03.10_15 | 16 | 0.52% | 9 | 0.20% |
| (Sub)acute lower backpain / radicular syndrome | 03.89_075 | 17 | 0.51% | 17 | 0.13% |
| Inguinal/Femoral hernia | 03.03_121 | 18 | 0.50% | 11 | 0.17% |
| Trigger finger release | 03.04_331 | 19 | 0.50% | 22 | 0.10% |
| Epistaxis | 03.02_35 | 20 | 0.45% | 27 | 0.09% |
| Meniscus tear | 03.05_1805 | 21 | 0.45% | 20 | 0.11% |
| Malignant breast neoplasm | 03.03_318 | 22 | 0.44% | 18 | 0.13% |
| Chronic superficial venous pathology/varices | 03.03_428 | 23 | 0.44% | 15 | 0.15% |
| OMA, OME, tuba dysfunction | 03.02_13 | 24 | 0.44% | 28 | 0.09% |
| Naevus, small lipoma, atheroma | 03.03_171 | 25 | 0.41% | 16 | 0.15% |
| Eczema, others | 03.10_08 | 26 | 0.39% | 58 | 0.05% |
| Impulse- and conduction disorders | 03.20_404 | 27 | 0.38% | 26 | 0.09% |
| Peripheral neuropathy (incl PHN) | 03.89_130 | 28 | 0.37% | 29 | 0.09% |
| Sinusitis | 03.02_36 | 29 | 0.34% | 41 | 0.07% |
| Incontinence/prolapse | 03.07_G25 | 30 | 0.32% | 32 | 0.08% |
